# Comparative transcriptome analysis of roots, stems and leaves of *Isodon amethystoides* reveals candidate genes involved in Wangzaozins biosynthesis

**DOI:** 10.1186/s12870-018-1505-0

**Published:** 2018-11-08

**Authors:** Fenglan Zhao, Mengchu Sun, Wanjun Zhang, Chunli Jiang, Jingtong Teng, Wei Sheng, Mingzhi Li, Aimin Zhang, Yongbo Duan, Jianping Xue

**Affiliations:** 1 Key Laboratory of Resource Plant Biology of Anhui Province, College of Life Sciences, Huaibei Normal University, Huaibei City, China; 2 Genepioneer Biotechnologies Co. Ltd, Nanjing City, China

**Keywords:** *Isodon amethystoides* (ben-th) cy Wu et Hsuan, Transcriptome, de novo assembly, Tetracyclic diterpenoid, Wangzaozins

## Abstract

**Background:**

*Isodon amethystoides* (Ben-th) Cy Wu *et* Hsuan is an important traditional medicinal plant endowed with pharmacological properties effective in the treatment of various diseases, including pulmonary tuberculosis. The tetracyclic diterpenoids, Wangzaozins (Wangzaozin A, glaucocalyxin A, glaucocalyxin B), are the major bioactive compounds of *I. amethystoides*. However, the molecular information about the biosynthesis of these compounds still remains unclear.

**Results:**

An examination of the accumulated levels of Wangzaozins in *I. amethystoides* revealed considerable variations in the root, stem, and leaf tissues of this plant, indicating possible differences in metabolite biosynthesis and accumulation among various tissues. To better elucidate the tetracyclic diterpenoid biosynthesis pathway, we generated transcriptome sequences from the root, stem, and leaf tissues, and performed de novo sequence assembly, yielding 230,974 transcripts and 114,488 unigenes, with average N50 lengths of 1914 and 1241 bp, respectively. Putative functions could be assigned to 73,693 transcripts (31.9%) based on BLAST searches against annotation databases, including GO, KEGG, Swiss-Prot, NR, and Pfam. Moreover, the candidate genes involving in the diterpenoid biosynthesis, such as CPS, KSL, were also analyzed. The expression profiles of eight transcripts, involving the tetracyclic diterpenoid biosynthesis, were validated in different *I. amethystoides* tissues by qRT-PCR, unraveling the gene expression profile of the pathway. The differential expressions of *ISPD*, *ISPF* and *ISPH* (MEP pathway), and *IaCPS* and *IaKSL* (diterpenoid pathway) candidate genes in leaves and roots, may contribute to the high accumulation of Wangzaozins in *I. amethystoides* leaves.

**Conclusion:**

The genomic dataset and analyses reported here lay the foundations for further research on this important medicinal plant.

**Electronic supplementary material:**

The online version of this article (10.1186/s12870-018-1505-0) contains supplementary material, which is available to authorized users.

## Background

The herbaceous genus *Isodon* (formerly *Rabdosia*), in the family Lemiaceae, contains a number of traditional medicinal plants that have a long history in traditional popular prescription in China. There are approximately 150 species of *Isodon*, nearly 70% of which are distributed in tropical and subtropical Asia [1, 2]. Many *Isodon* species have been shown to be effective in treating different illness, including *I. japonicus* for bleomycin-induced pulmonary fibrosis [3], *I. eriocalyx* for autoimmune encephalomyelitis amelioration [4], *I. phyllostachys* for its antiphlogistic and antibiotic actions [5], and *I. rubescens* for its anticancer, anti-inflammatory, and antibacterial properties [6]. *Isodon amethystoides* (Ben-th) Cy Wu *et* Hsuan, referred to as “Wang Zao Zi” and “Xiang Cha Cai” in China, is known for its broad applications as a folk remedy for abscesses, swollen sores, and tumors [7]. Unlike other traditional Chinese medicines needing concerted application, *I. amethystoides* is commonly used as a single drug for therapeutic purposes. Its ethanol extracts have shown a broad spectrum of antimicrobial efficacy, and in the 1980s were investigated as “Wang Zao Zi Soap” or “Wang Zao Zi Injection” for clinical trials in the Huaibei region of China [8]. The total healing rate reached 94% from 200 clinical cases with pneumonia, lung abscess, and pulmonary tuberculosis, thereby demonstrating its potential pharmacological value when used as a simple preparation.

Wangzaozins, including Wangzaozin A, glaucocalyxin A (Wangzaozin B), glaucocalyxin B (Wangzaozin C), are *ent*-kaurane-type tetracyclic diterpenoids isolated from Wangzaozi [9, 10]. Having the same basic carbon skeleton, the three compounds differ in modified groups. Wangzaozin A has a hydroxyl on C-3 where it is a ketone in glaucocalyxin A, and glaucocalyxin B is the acetylated structure of glaucocalyxin A [10]. Pharmacological studies have identified the bioactivities of Wangzaozins. For example, glaucocalyxin A has been widely studied for certain important biological activities, including antitumor [11], apoptosis induction [12, 13], antibacterial, anti-oxidative, anticoagulative, antithrombotic, antifibrotic, immune, and antineuroinflammatory activities [5, 14, 15]. Wangzaozin A, specifically isolated from Wang Zao Zi and not from other *Isodon* species, has been proved to be effective in the treatment of pneumonia, lung abscess, and pulmonary tuberculosis when applied independently.

In plants, tetracyclic diterpenoids are synthesized via the plastid methylerythritol phosphate (MEP) pathway [16, 17]. The biosynthesis initiates with the reaction between glyceraldehyde 3-phosphate and pyruvate, which generates IPP and DMAPP. Both IPP and DMAPP can be converted into geranyl diphosphate (GPP), which generates geranylgeranyl-PP (GGPP) to further synthesize diterpenoids. GGPP is then converted to tetracyclic diterpenoids by *ent-*copalyl diphosphate synthase (CPS), and *ent-*kaurene synthase (KS) through sequential steps. For *Isodon* species, Li et al. [18] reported the role of two *IeCPS* genes in *ent-*copalyl diphosphate synthesis in *I. eriocalyx* via homologous cloning. Very recently, the diterpene synthases (diTPSs) including CPS and KSL have been identified in *I. rubescens* [19, 20].

DNA sequence information is a prerequisite for elucidating the molecular mechanism underlying diterpenoid biosynthesis. For non-sequenced medicinal plants, RNA-seq provides an efficient tool for us to generate large amount of omics data. For *Isodon* species, the transcriptomic data were reported in *I. rubescens* [6, 21], but it has not been recorded in *I. amethystoides* and other species yet. In the present study, we report transcriptome databases from the root, stem, and leaf tissues of one-year-old *I. amethystoides* plants. Through comprehensive analysis of the transcriptome data, we identified thousands of genes, including a set of putative genes involved in the diterpenoid biosynthesis pathway and a number of transcription factors (TFs). Moreover, we analyzed the transcripts involving tetracyclic diterpenoid Wangzaozins biosynthesis in different tissues. This work lays the foundations for functionally elucidating the genes involved in Wangzaozins biosynthesis, and molecular regulation of the biosynthesis of bioactive compounds in *I. amethystoides*.

## Results

### Tetracyclic diterpenoid accumulation

**Fig. 1:**
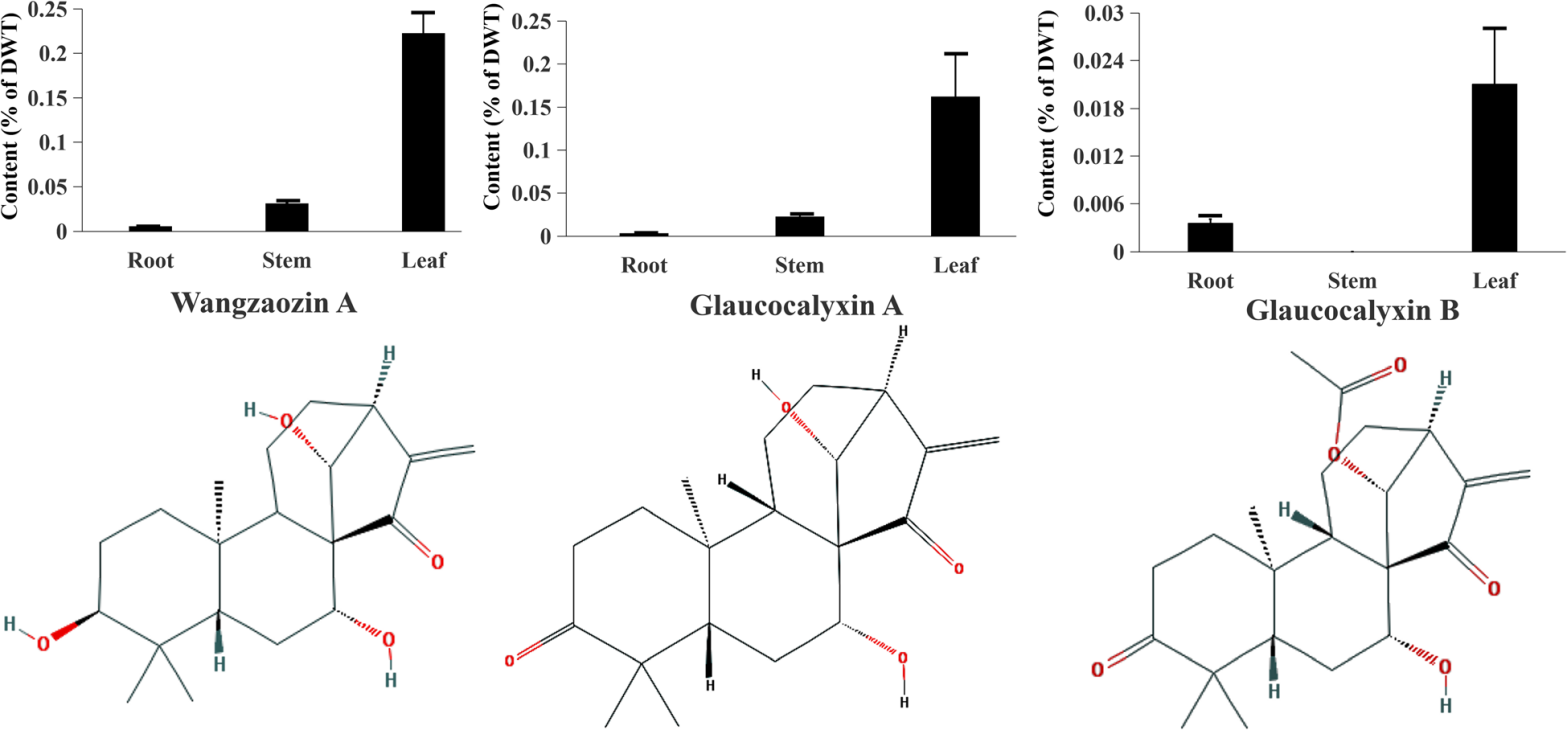
The content of Wangzaozin A, glaucocalyxin A, and glaucocalyxin B in different tissues

To examine the distribution pattern of tetracyclic diterpenoids, we determined the contents of three major bioactive tetracyclic diterpenoid compounds, Wangzaozin A, glaucocalyxin A, and glaucocalyxin B, in three tissues (root, leaf, and stem) of *I. amethystoides*. The three compounds have similar chemical structures, but differ in the structure of modified groups. The highest content of Wangzaozin A was observed in leaves, as high as 0.22% of DWT, followed by 0.031% in stems, which were 40- and 6-fold higher than that in roots, respectively. The highest accumulation of glaucocalyxin A was also found in leaves (0.16% of DWT), followed by stems, which were 48- and 7-fold higher than that in roots, respectively (Fig. 1). The content of glaucocalyxin B in leaves was 6-fold higher than that in roots, whereas no glaucocalyxin B was detected in stems. Due to the distinct distributions of the major tetracyclic diterpenoids, all three tissues (root, stem, and leaf) were selected as experimental materials for studying the tetracyclic diterpenoid biosynthesis pathway via comparative transcriptome analysis.

### Illumina sequencing and read assembly

To generate a transcriptome database for *I. amethystoides*, nine RNAseq libraries were constructed from the root, stem, and leaf tissues with three biological replicates. A total of 65.28 Gb clean data was recovered after trimming to remove adaptors, primer sequences, poly-A tails, and short and low-quality sequences. A range of 71 to 74 million for each tissue was obtained (Additional file 1: Table S2). Using the high-quality reads, 132,720 transcripts were assembled, with an average length of 1209 bp and N50 length of 1914 bp. Finally, 114,488 unigenes were identified. The total accumulated size of the assembled unigenes was approximately 98 Mb, with an N50 length of 1241 bp and an average size of 858 bp. There were 42,851 (43.419%) unigenes in the size range 300–500 bp, 30,335 (30.737%) at 501–1000 bp, 14,572 (14.765%) at 1000–2000 bp, and 10,933 (11.078%) > 2000 bp (Additional file 2: Figure S1). The correlation indices between repeated samples were ≈0.9 (Additional file 3: Table S3, Additional file 4: Figure S2), suggesting that the RNA-seq results are credible.

### Functional annotation

The *I. amethystoides* transcripts were annotated by BLAST searches against several public databases. A statistical summary of these annotations is listed in Additional file 5: Table S4. Among the 114,488 unigenes, 25,101 (21.92%) could be annotated in COG, 46557 (40.67%) in GO, 17527 (15.31%) in KEGG, 40517 (35.39%) in KOG, 47026 (41.08%) in Pfam, 47,100 (41.14%) in Swiss-Prot, and 72,165 (63.03%) in NR databases. Collectively, 73,693 transcripts (64.37%) could be assigned at least one putative function from one of these databases. Functional annotations of the assembled transcripts with plant NRDB demonstrated that the majority of these are homologous to those in species including *Sesamum indicum*, *Erythranthe guttata*, and *Vitis vinifera* (Additional file 6: Figure S3). The remaining 35.63% of the transcripts had no significant protein matches in these databases. These non-matched transcripts may be novel or diverse proteins and long non-coding RNAs in *I. amethystoides*, or could be derived from less conserved 3′ or 5′ untranslated regions of the genes.

Various TF families, including the MYB, WRKY, and bHLH families, have been confirmed to be involved in secondary metabolite biosynthesis [22, 23]. Transcriptome analysis of *I. amethystoides* revealed that 1373 unigenes (2%) encode putative TFs that can be classified into 55 TF families (Additional file 7: Table S5). Members of the C2H2 TF family were the most abundant (128, 9.32%), followed by those in the ERF (113, 8.23%), bHLH (101, 7.36%), bZIP (97, 7.06%), MYB-related (74, 5.39%), and MYB (66, 4.81%) families (Additional file 8: Figure S4). Identification of this large set of TFs, along with their expression profiling in individual tissues, provides a rich resource for further characterization of specific TFs in various biochemical pathways in *I. amethystoides*.

When GO was used to classify gene functions, 46,557 transcripts could be assigned to one or more GO terms within three domains and 51 functional categories (Additional file 9: Figure S5, Additional file 10: Table S6). Within the cellular component domain, the three most enriched categories were “cell part” (23,604, 50.70%), “cell” (23,485, 50.44%), and “organelle” (18,688, 40.14%). In the molecular function domain, the three most matched categories were “catalytic activity” (25,576, 54.93%), “binding” (22,564, 48.47%), and “transporter activity” (3134, 6.73%). In the biological process domain, the three most common categories were “metabolic process” (33,227, 45.09%), “cellular process” (26,914, 36.52%), and “single-organism process” (22,923, 31.11%). The most commonly assigned functional categories in each domain were consistent with the results from other *Isodon* species [24, 25].

**Fig. 2:**
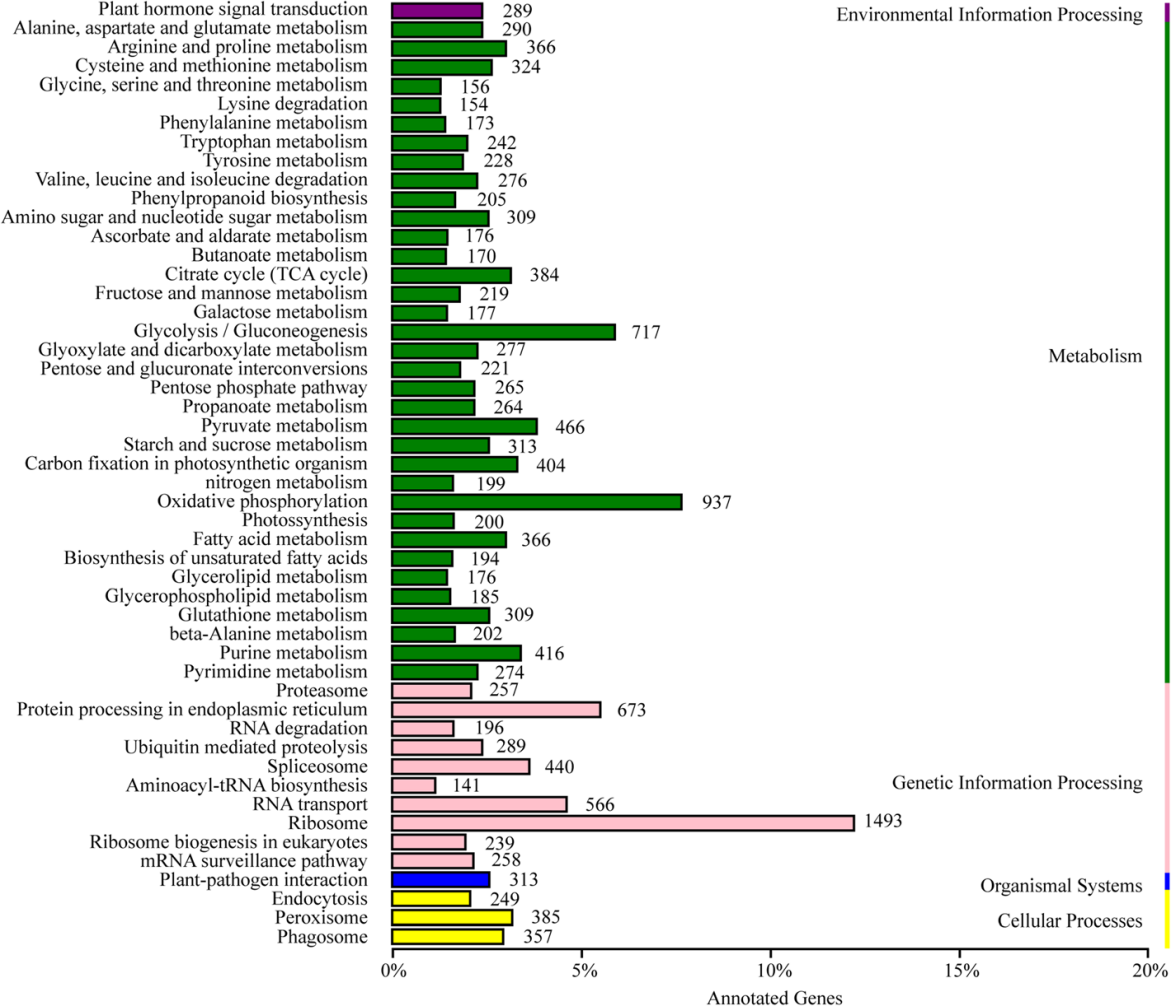
KEGG pathway classification map. Genes were divided into five branches according to corresponding biological pathways

The assembled genes were assigned to the biological pathways described in KEGG to better understand the functions in specific metabolic pathways in *I. amethystoides* (Fig. 2). To systematically analyze inner cell metabolic pathways and complex biological behaviors, the annotated coding region sequences were mapped to the reference canonical pathways in the KEGG pathway database. As a consequence, 17,527 transcripts (15.31%) could be assigned to five main categories: “metabolism,” “genetic information processing,” “environmental information processing,” “cellular process,” and “organismal systems,” with 50 sub-categories and 124 pathways (Fig. 2, Additional file 11: Table S7). These enzymes have assigned functions in 23 secondary metabolic pathways in KEGG (Table 1). Among these, 230 transcripts encode key enzymes involved in the pathways for tetracyclic diterpenoid biosynthesis, including the synthesis of the tetracyclic diterpenoid backbone (134 transcripts), monoterpenoids (2 transcripts), diterpenoids (29 transcripts), sesquiterpenoid (1 transcript), and other terpenoids (66 transcripts). Nine transcripts with high homology with GA oxidase sequences. Two hundred and eighty-eight transcripts were related to genes involved in the flavonoid biosynthesis pathway, including the phenylpropanoid (205 transcripts), flavonoid (64 transcripts), and flavone and flavonol (19 transcripts) biosynthesis pathways. Ninety transcripts were associated with alkaloid biosynthesis, including indole alkaloid biosynthesis (4 transcripts), isoquinoline alkaloid biosynthesis (38 transcripts), and tropane, piperidine, and pyridine alkaloid biosynthesis (48 transcripts). Identification and future characterization of transcripts involved in these different metabolic pathways will help us to better understand their functions in the biosynthesis of active compounds in *Isodon* plants.

**Table 1:** Secondary metabolism pathways in *Isodon amethystoides*

Pathway ID	Pathways	Functional categories	Unique gene number
ko00100	Steroid biosynthesis	21	74
ko00130	Ubiquinone and other terpenoid-quinone biosynthesis	17	66
ko00230	Purine metabolism	91	416
ko00232	Caffeine metabolism	9	11
ko00400	Phenylalanine, tyrosine, and tryptophan biosynthesis	36	89
ko00860	Porphyrin and chlorophyll metabolism	32	130
ko00900	Terpenoid backbone biosynthesis	28	134
ko00901	Indole alkaloid biosynthesis	4	4
ko00902	Monoterpenoid biosynthesis	2	2
ko00903	Limonene and pinene degradation	6	94
ko00904	Diterpenoid biosynthesis	8	38
ko00905	Brassinosteroid biosynthesis	6	6
ko00906	Carotenoid biosynthesis	24	78
ko00908	Zeatin biosynthesis	7	46
ko00909	Sesquiterpenoid biosynthesis	1	1
ko00940	Phenylpropanoid biosynthesis	23	205
ko00941	Flavonoid biosynthesis	16	64
ko00942	Anthocyanin biosynthesis	1	1
ko00944	Flavone and flavonol biosynthesis	7	19
ko00945	Stilbenoid, diarylheptanoid, and gingerol biosynthesis	6	49
ko00950	Isoquinoline alkaloid biosynthesis	8	38
ko00960	Tropane, piperidine, and pyridine alkaloid biosynthesis	9	48
ko00965	Betalain biosynthesis	1	1

### Differential gene expression analysis

**Fig. 3:**
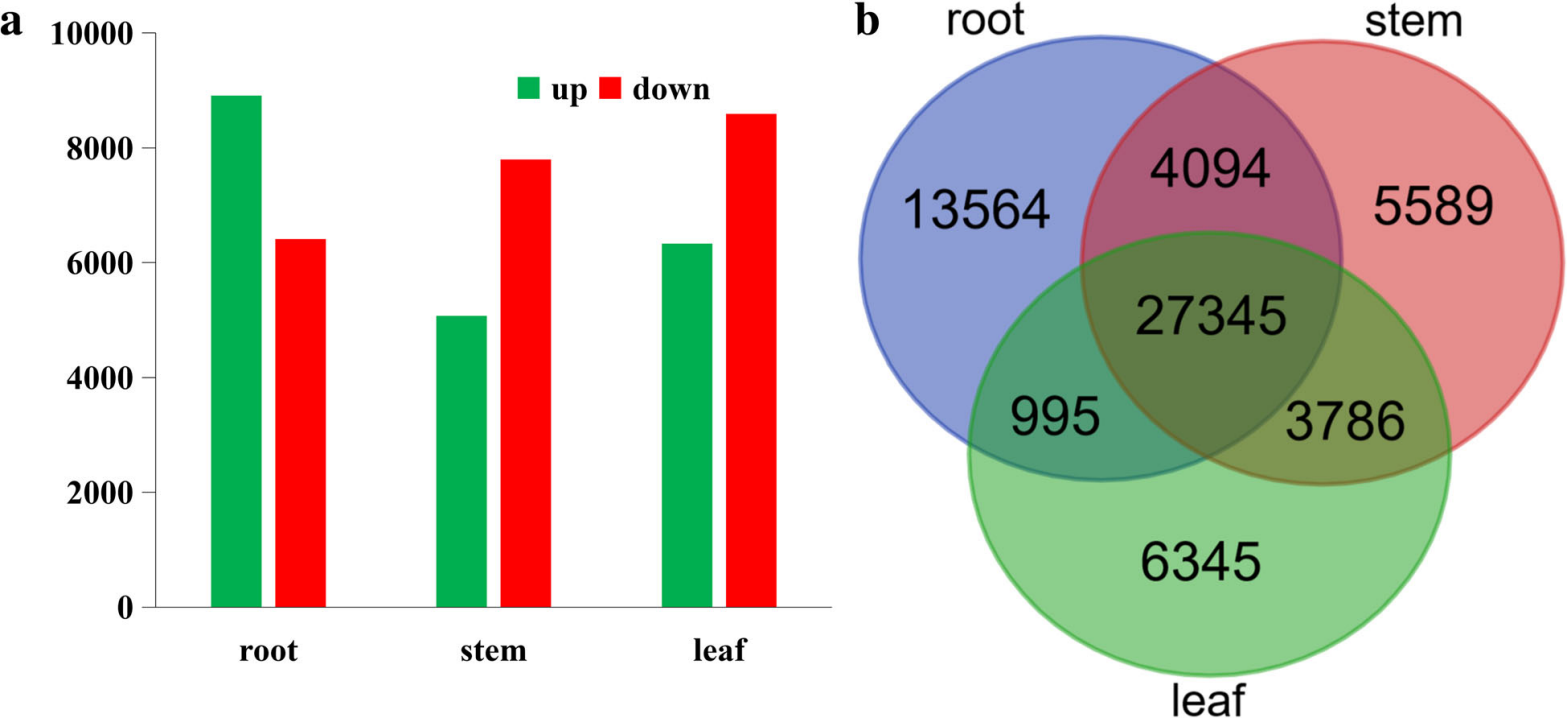
Differential expression analysis of transcripts. **a** The number of differentially expressed transcripts (*P* < 0.05 and > 2 folds change) in each tissue compared with two other tissues. **b** Venn diagram representing the number of DETs among *Isodon amethystoides* tissues

To investigate the differential gene expression among different tissues, the high-quality reads from individual samples were mapped onto the *I. amethystoides* transcriptome using CLC Genomics Workbench (v8.0.2). DEGs were identified (Additional file 12: Table S8) using the DESeq package with FDR ≤ 0.01 and at least a 2-fold expression change among different tissues [26]. The number of DEGs was highest between the root and leaf, and lowest between the stem and leaf. Furthermore, the DEGs in one tissue with respect to those in the other two tissues were also investigated. Roots contained the largest number of most highly expressed transcripts, with 8906 transcripts being more abundant than in the other organs, while 6410 were less abundant (Fig. 3a). In contrast, in stems, 5072 genes were expressed at a higher level than in roots and leaves, while 7792 genes were expressed at a lower level. In addition, a total of 6354 transcripts exhibited tissue-specific expression, with 1127, 760, and 4467 transcripts being specifically expressed in root, stem, and leaf, respectively (Fig. 3b). Among the identified DEGs, 1373 encoded TFs, some of which were specifically expressed in the root, stem, and leaf (Additional file 12: Table S8). Future characterization of specific TFs is required for a better understanding of the gene expression profiles and regulation in the various biochemical pathways in *I. amethystoides*.

**Fig. 4:**
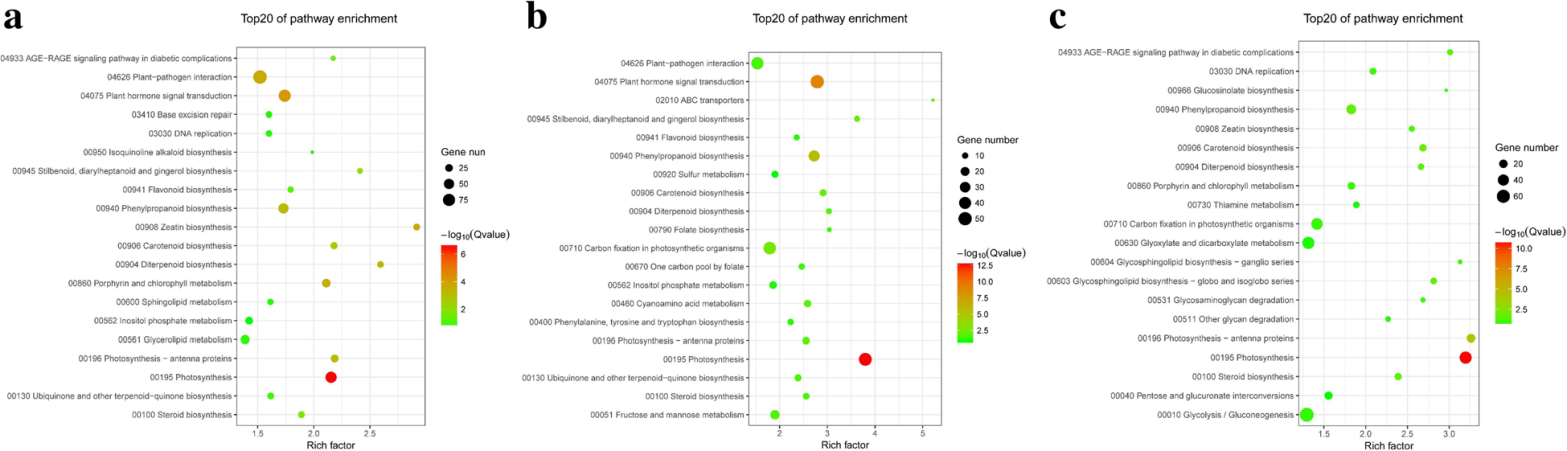
KEGG enrichment analyses with the DEGs among root, leaf, and stem. **a** Between root and leaf; **b** between leaf and stem; **c** between root and stem

To explore the possible genes regulating the distinct distribution of Wanzaozins, we further performed KEGG enrichment analyses with the DEGs among root, leaf, and stem. These DEGs significantly enriched in a number of pathways. Between root and leaf, photosynthesis, plant hormone signal transduction, zeatin biosynthesis, carotenoid biosynthesis, photosynthesis - antenna proteins, diterpenoid biosynthesis, porphyrin and chlorophyll metabolism, and phenylpropanoid biosynthesis, are the first eight pathways showing the greatest differential expression (Fig. 4a). Between leaf and stem, the eight pathways showing significant enrichment were photosynthesis, plant hormone signal transduction, phenylpropanoid biosynthesis, starch and sucrose metabolism, carotenoid biosynthesis, cyanoamino acid metabolism, ABC transporters, and phenylalanine metabolism (Fig. 4b). Between root and stem, photosynthesis, photosynthesis - antenna proteins, phenylalanine metabolism, phenylpropanoid biosynthesis, steroid biosynthesis, carotenoid biosynthesis, diterpenoid biosynthesis were the eight categories showing significant enrichment (Fig. 4c). The pathways of primary metabolism, including photosynthesis, amino acid metabolism, and starch and sucrose metabolism, which are essential to plant growth, were common in all three tissues. With the exception these common pathways, the enriched secondary metabolic pathways, including diterpenoid and flavonoid biosynthesis, were also detected between different tissues, particularly between roots and above-ground parts (Additional file 13: Table S9), indicating the possible distinct distribution of secondary metabolites among various tissues.

### Putative genes involved in the tetracyclic diterpenoid biosynthesis

Totally 29 transcripts classified into 12 enzyme categories were identified by using transcriptome data to examine the KEGG database (Additional file 14: Table S10). NCBI blast showed that these candidate transcripts displayed 67—99% homology with their respective best matches.

**Fig. 5:**
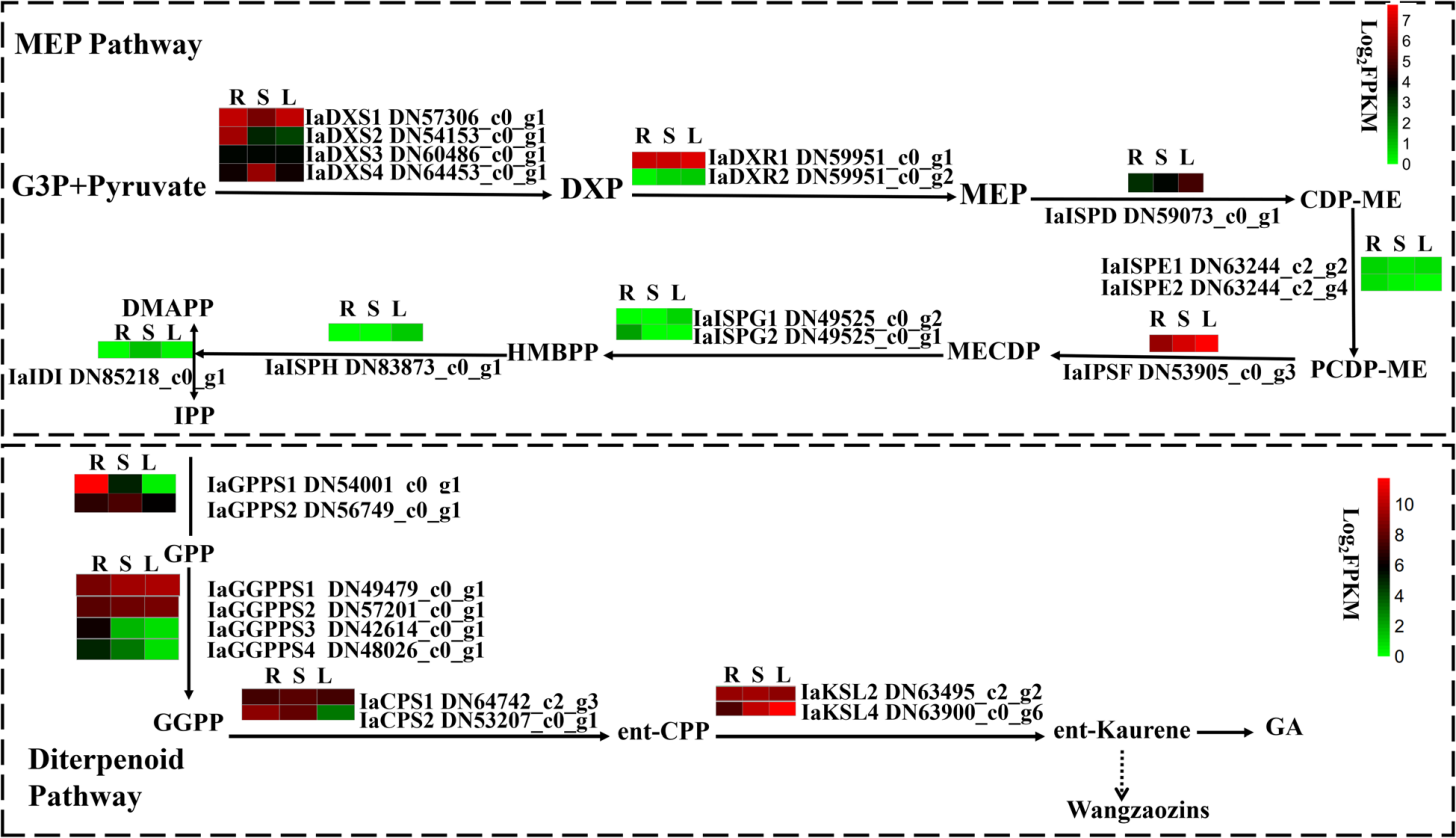
Schematic representation of the proposed diterpenoid biosynthesis pathway and differential expression of genes identified. The transcriptomic data (log_2_FPKM) for each DEG represent the expression in root (R), stem (S), and leaf (L) on heat map from the left

The putative pathway of tetracyclic diterpenoids Wangzaozins biosynthesis was proposed (Fig. 5). In the MEP pathway, 14 transcripts were identified, including those of four 1-deoxy-d-xylulose-5-phosphatesynthases (DXS) and two 1-deoxy-d-xylulose-5-phosphate reductoisomerases (DXR). DXS and DXR promote the generation of 2-C-methyl-derythritol 4-phosphate (MEP) from the substrate d-glyceraldehyde 3-phosphate and pyruvate. Of the four DXS genes, IaDXS1 expressed similarly in all three tissues, whereas IaDXS4 showed the highest expression in stem. Both IaDXR1 and IaDXR2 displayed a constitutive expression in three tissues, and IaDXR1 had a remarkably higher expression than IaDXR2. These suggest that IaDXS1, IaDXS4, and IaDXR1 are important for the generation of MEP and deserve further functional characterization. MEP further reacts under the catalysis of following enzymes. In our transcriptomic dataset, only one transcript for 2-C-methyl-d-erythritol 4-phosphate cytidylyltransferase (ISPD), 1-hydroxy-2-methyl-2-(E)-butenyl 4-diphosphate reductase (ISPF), and 1-hydroxy-2-methyl-2-(E)-butenyl 4-diphosphate reductase (ISPH) each was identified, and their expressions were 2 to 3 - fold higher in leaf than root, inferring that these three genes may be single copy genes. There are two transcripts for both IaISPE and IaISPG, which displayed constitutive expressions or predominant expression in one of the three tissues. These genes may lead to high efficiency of tetracyclic diterpenoid substrate biosynthesis in leaf of *I. amethystoides*.

The tetracyclic diterpenoid biosynthesis involves geranyl diphosphate synthase (GPPS), geranylgeranyl diphosphate synthase (GGPPS), *ent-*copalyl diphosphate synthase (CPS) and *ent-*kaurene synthase (KSL). In total, 15 transcripts were found to be involved in this pathway. Two GPPS and four GGPPS transcripts were identified in our dataset. IaGPPS1 was expressed predominantly in root and rarely detectable in leaf, while IaGPPS2 showed the highest expression in stem. IaGGPS1 was expressed at a considerably higher level in above ground parts than roots, whereas IaGGPS2 showed a similar expression among three tissues.

**Fig. 6:**
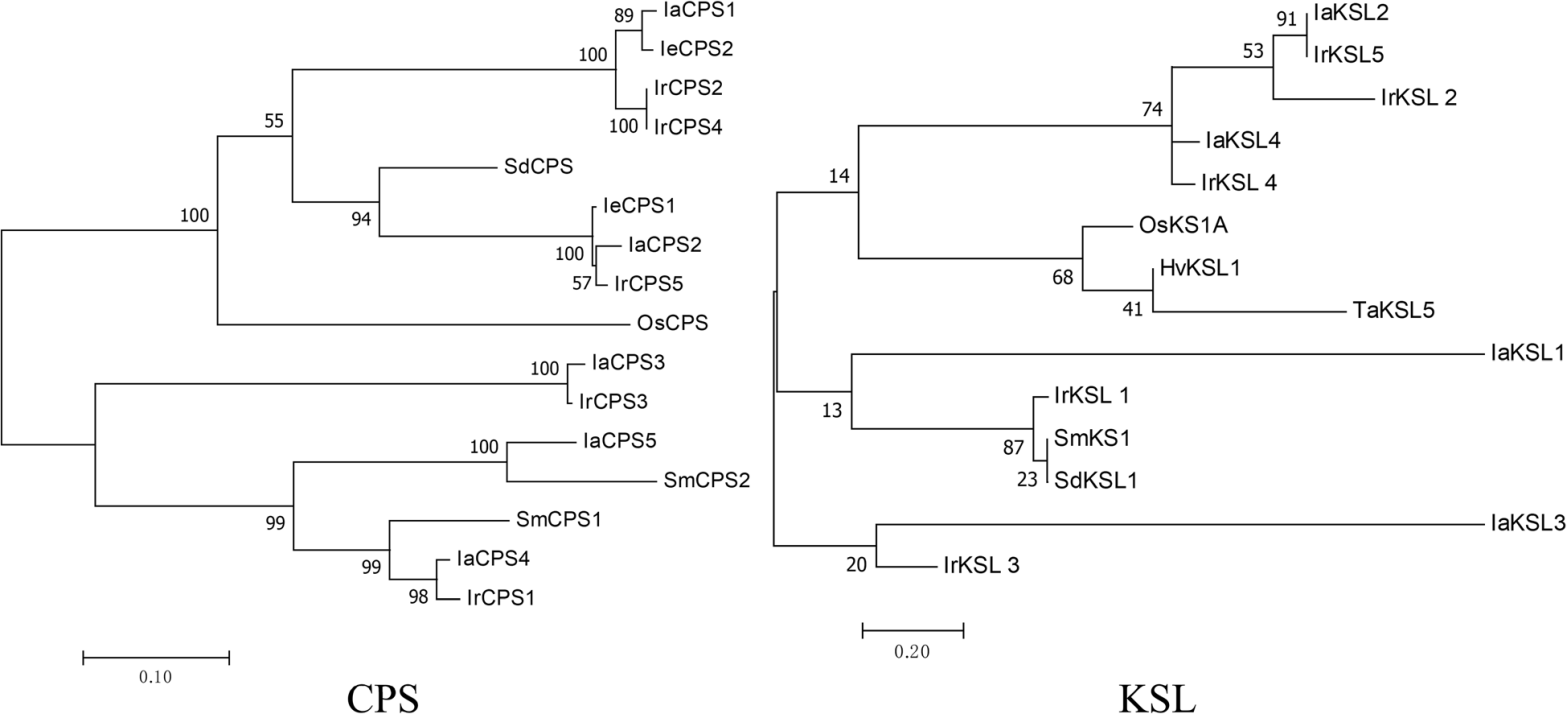
Phylogenetic analysis of *Isodon amethystoides* CPS and KSL candidate genes by the maximum likelihood method with MEGA software. *I*. *amethystoides* CPS and KSL transcripts listed in Additional file 14: Table S10 were blasted in NCBI to obtain the CDS information for phylogenetic analysis. IeCPS1, *I*. *eriocalyx ent-*copalyldiphosphate synthase (AEP03177); IeCPS2, *I*. *eriocalyx ent-*copalyldiphosphate synthase (AEP03175); IrCPS1, *I. rubescens ent-*copalyldiphosphate synthase (KU180499); IrCPS3, *I. rubescens* copalyl diphosphate synthase 3 (APJ36373); IrCPS4, *I. rubescens* copalyl diphosphate synthase 4 (APJ36374); IrCPS5, *I. rubescens* copalyl diphosphate synthase 5 (APJ36375); SmCPS1, *Salvia miltiorrhiza* copalyl diphosphate synthase 1 (KC814639); SmCPS2, *S. miltiorrhiza* copalyl diphosphate synthase 2 (AHJ59322); SdCPS, *Scoparia dulcis ent-*copalyldiphosphate synthase (BAD91286); OsCPS, *Oryza sativa ent-*copalyldiphosphate synthase (BAD424492); IrKSL1, *I. rubescens ent-*kaurene synthase (KUI180504); IrKSL2, *I. rubescens ent-*kaurene synthase (KUI180505); IrKSL3, *I. rubescens ent-*kaurene synthase (KUI180506); IrKSL4, *I. rubescens ent-*kaurene synthase (KX580633); IrKSL5, *I. rubescens ent-*kaurene synthase (KX580635); TaKSL5, *Triticum aestivum* nerolidol synthase (BAL41692); OsKSL1, *Oryza sativa ent-*kaurene synthase (AAQ72559); HvKSL1, *Hordeum vulgare ent-*kaurene synthase (AAT49066). Numbers on branches indicate the bootstrap percentage values calculated from 1000 bootstrap replicates

With regard to CPS, an important enzyme in the diterpenoid pathway, 5 transcripts were found in *I. amethystoides*. IaCPS4 showed constitutive expression among three tissues, while IaCPS1 had a higher expression in stem than other tissues. In the dataset we found one CPS transcript, IaCPS3 (DN34471_c0_g1), containing the complete open reading frame, was expressed predominantly in leaves. Phylogenetic analysis indicated that IaCPS1 fell into a same clade with IeCPS2, and it also has a high similarity with IrCPS4 (Fig. 6). IeCPS2 and IrCPS4 have been determined to be involved in pharmacologically active *Isodon* diterpenoids biosynthesis [18, 19]. Therefore, the CPS candidate genes, especially IaCPS1 (DN64742_c2_g3), may have important roles in Wangzaozins biosynthesis.

For *ent-*kaurene synthase, we detected 4 possible transcripts. Except for IaKSL2, which showed a similar expression level in all three tissues, the other three were expressed at a considerably higher level in leaves and stems. Particularly, IaKSL4 was highly expressed in leaf and stem. These KLS candidate transcripts shared high homologies with known counterparts in other species (Fig. 6). IaKSL2 and IaKSL4 were closely related to IrKSL5 and IrKSL4, respectively. IrKSL2 and IrKSL5 have been shown to react with *ent-*CPP [19]. The results indicate that KSL genes (IaKSL2 and IaKSL4) having a high or predominant expression, may be a cause of accumulation of Wangzaozins in *I. amethystoides* leaves.

The transcriptomic data for different tissues would be helpful for obtaining the CDS of putative genes involved in Wangzaozins biosynthesis pathways and unraveling their family members and functions in *I. amethystoides*.

### Expression patterns of gibberellin (GA) biosynthesis candidate genes among different tissues

**Fig. 7:**
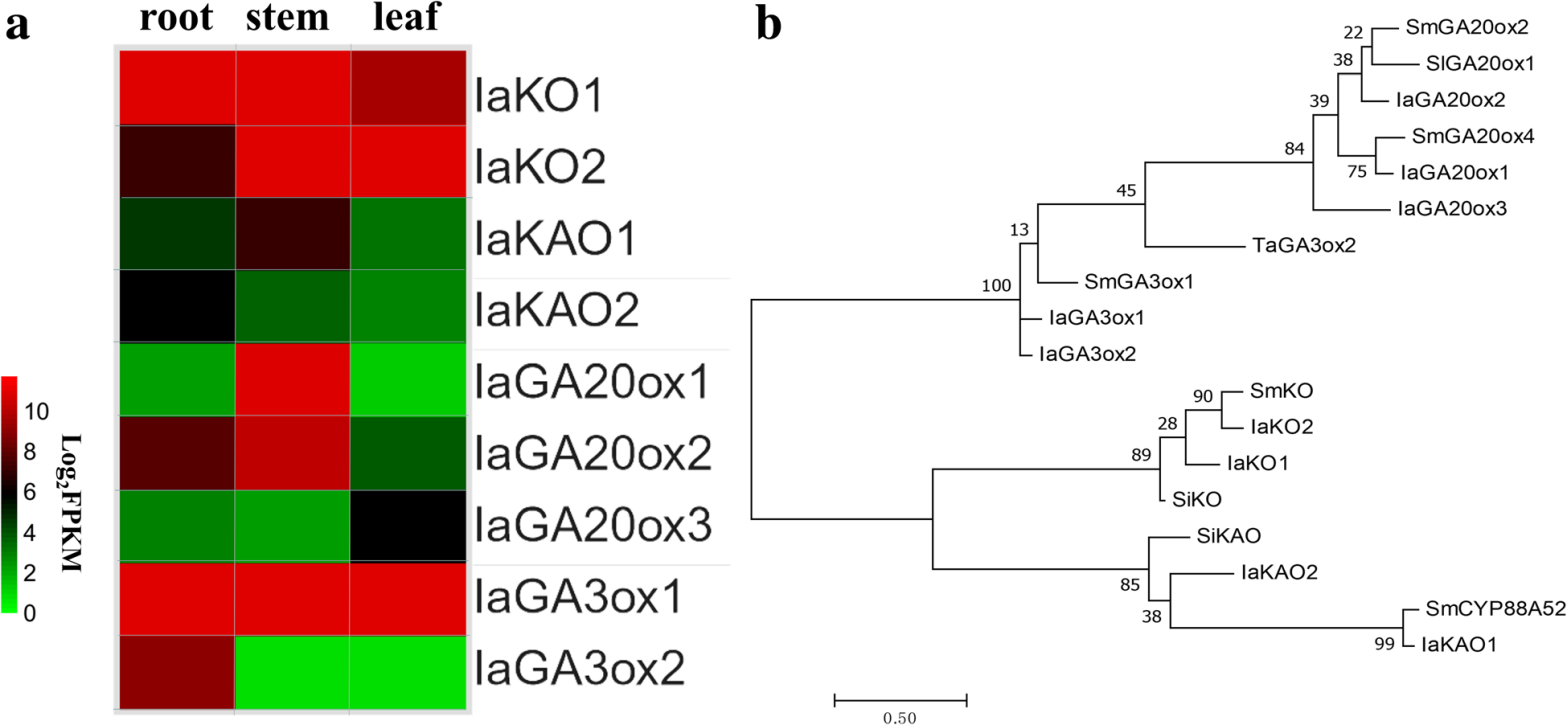
Expression of candidate genes associated with gibberellins biosynthesis. **a** Schematic representation of gibberellin biosynthesis associated transcripts by heatmap based on FPKM values; **b** phylogenetic analysis of potential genes by the maximum likelihood method with MEGA software. IaKO1, DN62518_c0_g1; IaKO2, DN63069_c0_g1; IaKAO1, DN47083_c0_g1; IaKAO2, DN60719_c0_g6; IaGA20ox1, DN51838_c0_g1; IaGA20ox2, DN59747_c0_g2; IaGA20ox3, DN51642_c0_g1; IaGA3ox1, DN58138_c0_g2; IaGA3ox2, DN58138_c0_g1; SiKO, *Sesamum indicum ent-*kaurene oxidase (XP011074464); SmKO, *Salvia miltiorrhiza ent-*kaurene oxidase (ALR74628); SmCYP88A52, *S. miltiorrhiza* cytochrome P450 (AJD25209); SiKAO, *S. indicum ent-*kaurenoic acid oxidase 2 (XP020548506); SmGA20ox2, *S. miltiorrhiza* gibberellin 20-oxidase 2 (ALR74634); SmGA20ox4, *S. miltiorrhiza* gibberellin 20-oxidase 4; SmGA3ox1, *S. miltiorrhiza* gibberellin 3-oxidase 1 (ALR74631); SlGA20ox1, *Solanum lycopersicum* gibberellin 20-oxidase 1, (ABS84235); TaGA3ox2, *Triticum aestivum* gibberellin 3-oxidase 2 (AAZ94378). Numbers on branches indicate the bootstrap percentage values calculated from 1000 bootstrap replicates

Wangzaozins, belonging to *ent-*kaurane-type tetracyclic diterpenoids [9, 10], share the same requisite skeletal structure with gibberellin, as well as oridonin. Oridonin has been reported to have the same precursor of *ent-*kaurene with gibberellin [19, 27]. Therefore, *ent-*kaurene is thought to be the common substrate for biosynthesis of gibberellin and Wangzaozins. Because plant hormone signal transduction showed significant enrichment between root and leaf, and stem and leaf, we analyzed the expressions of GA biosynthesis genes among three tissues. Nine transcripts that are potentially involved in gibberellin biosynthesis were detected: three encoding gibberellin 20-oxidases (GA20ox), two gibberellin 3-oxidases (GA3ox) two *ent*-kaurene oxidases (KO) and two *ent*-kaurenoic acid oxidases (KAO) (Fig. 7a). Except for IaGA3ox1 being expressed consistently, all other genes showed differential expression patterns among three tissues. Phylogenetic analysis showed that these GA biosynthesis related candidate genes had a close genetic relationship with those reported in other *Isodon* species or dicots (Fig. 7b). The differential expressions of these candidate transcripts may lead to the difference in GA production among different tissues.

### qRT-PCR validation of candidate genes involved in Wangzaozins biosynthesis

**Fig. 8:**
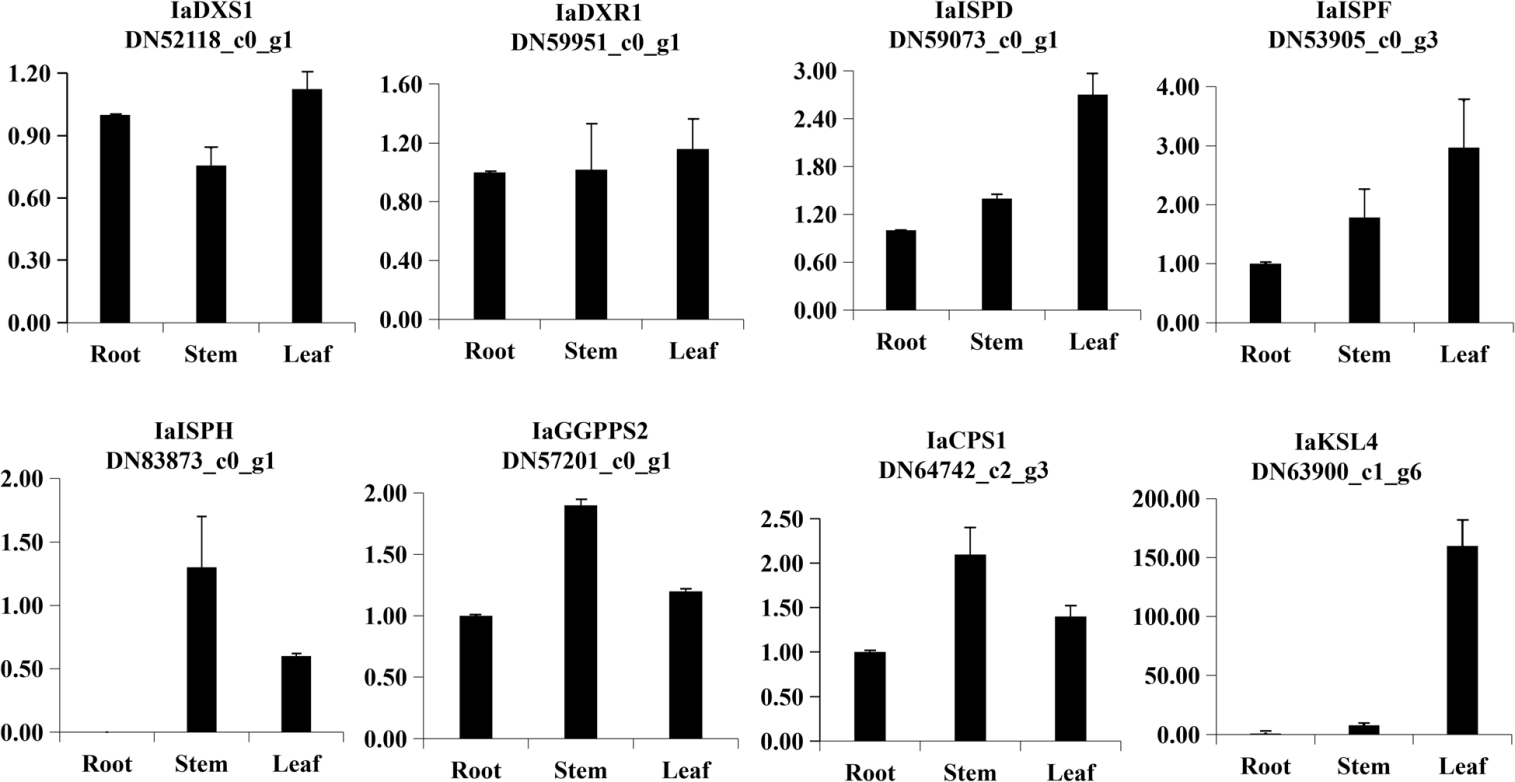
The expressions of eight genes with differential expression among various tissues

To validate the transcriptome analysis data and also to evaluate the differential expression profile among different tissues, we selected eight transcripts cognate to Wangzaozins biosynthesis for qRT-PCR analysis. The expressions of these transcripts based on FPKM value are shown in Fig. 8. After validation, it was found that all eight transcripts showed a similar expression pattern based on FPKM value, though there are some differences in expression folds. Both IaDXS1 and IaDXR1 showed a similar expression among three tissues. The single copy transcripts IaISPD, IaISPF and IaISPH had a predominant expression in leaf or stem. Interestingly, IaISPH was not detected in root by qRT-PCR. IaGGPPS2 and IaCPS1 showed an approximately 2-fold higher expression in stems than root. *IaKSL*4 was expressed predominantly in leaf, with a 159-fold increased expression compared to that in the root respectively. The qRT-PCR results of all eight genes were in a good agreement with the transcriptomic data. The results further confirm the high reliability of transcriptomic data from three replicates, which will be helpful for understanding the biosynthesis pathway of Wangzaozins in *I. amethystoides*.

## Discussion

Tetracyclic diterpenoids are a kind of 20-carbon (C_20_) isoprenoids that are endowed with broad-spectrum bioactivities. Their biosynthesis and accumulation are usually restricted to specialized tissues or cell types. Usually the specialized diterpenoids are stored in the place of their synthesis [28]. Therefore, the tissue-specific accumulations of tetracyclic diterpenoids are associated with differential gene expression rather than their translocation to specialized tissues [16].

For non-model species lacking a well-studied genetic background, de novo transcription analysis provides an effective means of generating comprehensive information for the discovery of novel genes, and construction of gene expression networks for specific tissues [29–31]. In this study, a total of 65.28 Gb clean data were obtained from nine RNA-seq libraries of root, stem, and leaf. De novo assembly generated 114,488 transcripts with a total accumulated size of ≈98 Mb. The assembled data had an average N50 length of 1241 bp, which is similar to that recorded previously in other non-model plants, such as *Andrographis paniculata* [32, 33] and *Raphanus sativus* [34].

Functional annotation indicated that 64.37% (73695) of the transcripts could be assigned to at least one putative function from one of the COG, KEGG, Pfam, Swiss-Prot, or NR databases. This indicates the good de novo assembly of the *I. amethystoides* transcriptomic data. In its relative species *Isodon reubescens*, Su et al. [6] generated a dataset of 44,626 unigenes from leaf transcriptome, and 57.54% (25677) were annotated in one of the databases. In this study, we obtained more unigenes (approximately three folds) in *I. amethystoides* by comparative analysis of root, stem and leaves, which provide more abundant genetic information for understanding the molecular mechanism of Wangzoazins biosynthesis.

On the basis of the large-scale transcriptome data, identification of candidate genes of transcription factors (TFs) or those encoding key enzymes is essential for elucidating the biosynthesis of tetracyclic diterpenoids in *I. amethystoides*. TFs, which generally occur as gene families, play key roles in regulating the expression of genes by specifically binding to the *cis*-regulatory elements in the promoter regions of target genes [35]. Approximately 2% (1373) of the assembled transcripts were TFs, which could be classified into 55 TF families, including the C2H2, bZIP, and bHLH families. The roles of TFs in regulating diterpenoids have been widely studied in crops [36, 37]. For the Lamiaceae family, bZIP and bHLH genes have been found to be involved in the regulation of tanshinone biosynthesis [38, 39]. The evidence suggest that functional characterization of TFs has the potential to increase tetracyclic diterpenoid production. However, there is no reports focusing on the role of TFs in regulating diterpenoid biosynthesis in *Isodon* species. Thus, the candidate TFs provide a large gene pool to mine important regulatory genes for Wangzaozins production.

For the biosynthesis of Wangzoazins, 29 transcripts, including 14 in the MEP pathway and 15 in the tetracyclic diterpenoid biosynthesis were identified in this study. *ISPD*, *ISPF*, and *ISPH* have been found in our RNA-seq dataset as a single copy, consistent with the conclusion in *Stevia rebaudiana* [16]. All the three transcripts had a 2 to 3- fold higher expression in leaf than root. Four DXS and two DXR genes had constitutive expressions or similar expression among three tissues, which implies that these genes may play roles both in primary and diterpenoid metabolisms. The GGPPS family has been reported to be encoded by two to 12 members in plants [40]. Four IaGGPPS candidate genes were found in the present study, of which IaGGPPS1 (DN49479_c0_g1) showed higher expressions in above ground parts compared to the root.

Five CPS transcripts were screened out from the RNA-seq dataset, with similar expression among the three examined tissues, or preferential expression in leaf, stem, or root. From these CPS transcripts, we identified one (IaCPS3, DN34471_c0_g1) containing the complete open reading frame. Phylogenetic analysis revealed that it shares a high amino acid identity with *I. rubescens* IrCPS3. IrCPS3 was expressed five-fold higher in stem than root and leaf [19]. Other CPS transcripts share a high homology with known CPS genes from other plant species. Particularly, *IaCPS*1 was placed in the same clade with *I. eriocalyx IeCPS*2. *IeCPS*2 has been shown to specifically involved in the biosynthesis of pharmacologically active *Isodon* diterpenoids rather than gibberellin [18], implying a potential role of *IaCPS*1 in the biosynthesis of Wangzaozins.

The *ent*-kaurene synthase (EC 4.2.3.19) is an enzyme that catalyzes the formation of *ent*-kaurene from *ent-*copalyl diphosphate by releasing diphosphate. From the RNA-seq dataset, we identified four kaurene synthase-like genes in *I. amethystoides*. *IaKSL*2 and *IaKSL*4 are closely related to IrKSL5 and IrKSL4 respectively. In *I. rubescens*, IrKSL5 and IrKSL4 are involved in the generation of *ent*-kaurene [19]. It suggests that *IaKSL*2 and *IaKSL*4 may be the potential genes to react with *ent*-CPP in *I. amethystoides*.

The functional diversification of diterpene syntheses has been discovered in *Salvia miltiorrhiza* [41] and *I. rubescens* [19]. Therefore, the candidate genes identified in this study will make an important contribution to molecular studies on the biosynthesis of tetracyclic diterpenoid biosynthesis in *I. amethystoides*.

Gibberellins are diterpenoids that are derived biosynthetically from *ent*-kaurene, as some pharmacological diterpenoids [18, 19, 27]. In this study, the expressions of 9 transcripts associated with gibberellin biosynthesis were analyzed using the transcriptomic data. Except for IaGA3ox1 being expressed similarly among the three tissues, all other genes showed differential expression patterns. This infers the possible difference in GA production among diverse tissues of *I. amethystoides*.

## Conclusion

We generated a comprehensive transcriptome assembly harboring genetic information for mass sequence data of *I. amethystoides*. These sequence data allowed us to identify and characterize the molecular functions of TFs and candidate transcripts associated with tetracyclic diterpenoid metabolic pathways, which provide insight into the biosynthesis of bioactive compounds in *I. amethystoides*. Particularly, twenty nine transcripts encoding key enzymes in tetracyclic diterpenoid biosynthesis were identified. The higher expressions of *IaISPD*, *IaISPE* and *IaISPF* (MEP pathway), and *IaCPS* (such as *IaCPS*1) and *IaKSL* (such as *IaKSL*2 and *IaKSL*4) candidate genes in leaves, may contribute to the high accumulation of Wangzaozins in *I. amethystoides* leaves. Intensive biochemical, enzymatic, physiological, and molecular studies on these candidate transcripts will be necessary to better understand the underlying regulatory mechanisms. Moreover, our study records the first genomics resource for *I. amethystoides* and lays the foundations for further improvements in this medicinal crop through genetic, genomic, and engineering methods.

## Methods

### Plant materials

One-year-old *Isodon amethystoides* (Ben-th) Cy Wu et Hsuan plants, identified by Prof. Jianping Xue (Huaibei Normal University), were collected from the Experimental Farm of Huaibei Normal University, Huaibei City, Anhui Province, P.R. China (33°16′N, 116°23′E, altitude, 340 m), in May 2015. Root, stem, and leaf samples were collected separately from randomly healthy plant individuals.

### Extraction and estimation of Wangzaozins

Each tissue was pooled from three plants, air-dried, and ground into a fine powder. One gram of powder was weighed into 20 mL of ethanol for ultrasonication extraction at room temperature for 1 h. The extract was collected by filtering and then dried via rotary evaporation. The yielded pellet was dissolved in methanol to an appropriate concentration.

Standard references of Wangzaozins (Wangzaozin A, glaucocalyxin A, and glaucocalyxin B) were purchased from Chengdu Purechem-standard Co., Ltd. (Chengdu, China). Each standard reference was dissolved in methanol to yield a 0.1 mg·mL^− 1^ solution.

An Agilent Technologies 1260 Infinity Series HPLC system equipped with a Thermo-C18 column (250 mm × 4.6 mm, 5.0 μm) was used for liquid chromatographic separations. The mobile phase consisted of 0.1% acetic acid in water (solvent A) and methanol (solvent B). The gradient elution was as follows: 0–6 min, linear from 40 to 45% B; 6–35 min, held at 45% B; 35–45 min, linear from 45 to 95% B. The flow rate was 1.0 mL/min. The injection volume was 10 μL. The column temperature was set at 30 °C and the sampler was set at 4 °C.

The contents of Wangzaozin A, glaucocalyxin A, and glaucocalyxin B in each tissue were quantified by calculating the peak area compared with those of the respective standards by linear regression. The content was presented as an average percentage of dry weight (DWT) from three replicates.

### RNA extraction and transcriptome sequencing

All plant samples were separately cut into small pieces, and 2.0 g (fresh weight) of each tissue pooled from three plants was used for RNA isolation. The experiments contained three biological replications, and thus nine plants were used for each tissue. Total RNA extraction and RNA integrity evaluation were performed according to a previously published protocol [42]. RNA sequencing libraries with an average insert length of 250 bp were sent Genepioneer Technologies Corporation (Nanjing, China) for sequencing on Illumina HiSeq4000 platform (Illumina Inc., USA).

### Data filtering and de novo assembly

The raw data of nine RNA sequencing reads were trimmed to remove the adaptor sequences, duplicated sequences, ambiguous reads (‘N’), and low-quality reads. The yielded clean reads were then used for sequence assembly using the Trinity program with default parameters [43]. Contigs were generated by combining the clean reads with a certain overlap and then clustered using TGICL software [44] to yield unigenes. Non-redundant unigenes were acquired by removing the redundancies (−l 40, −c 10, and -v 20).

### Functional annotation and classification

The unique sequences were annotated against public databases, including the NR (http://www.ncbi.nlm.nih.gov/), Swiss-Prot (http://www.expasy.ch/sprot), Clusters of Orthologous Groups (COG) (http://www.ncbi.nlm.nih.gov/COG/), and Pfam (http://pfam.xfam.org/) databases, using BLASTX (http://blast.ncbi.nlm.nih.gov/Blast.cgi) with an E value of 1e^− 5^ [45]. GO analysis was performed to obtain the Gene Ontology (GO) functional classifications [46]. KEGG classification maps were drawn based on the retrieved Kyoto Encyclopedia of Genes and Genomes (KEGG) Orthology (KO) information. The transcription factors (TFs) in the *I. amethystoides* transcriptome were identified by searching the plant transcription factor database PlantTFDB 4.0 with all assembled unigenes [47].

### Differentially expressed genes (DEGs)

The fragments per kb per million reads (FPKM) method was employed to quantify the transcript abundances [26]. The thresholds for the significance test were set as FDR value ≤0.01 and the absolute value of log2 ratio ≥ 1. Each set of differentially expressed unigenes was analyzed by hypergeometric Fisher exact test (*P* < 0.01) and Benjamini correction (FDR < 0.05) to construct the KEGG pathways.

### Verification of gene expression using qRT-PCR

Eight DEGs related to Wangzaozins biosynthesis were selected for quantitative real-time PCR analysis. The primer sequences used are listed in Additional file 15: Table S1. qRT-PCR was performed using the ABI7500 fast Real-Time PCR system (Applied Biosystems, USA) with a SYBR® Premix ExTaq™ Kit (Takara, Dalian, China). The *Actin* gene was used for normalization of expression level. Three biological replicates and three technical repeats were contained for each gene and sample. The 2^−ΔΔCT^ method was used to calculate the relative gene expression [48].

## Additional files

Additional file 1: **Table S2.** Overview of transcriptome sequencing and de novo assembly. (DOC 18 kb)

Additional file 2: **Figure S1.** Overview of transcriptome assembly showing size distribution. (TIF 714 kb)

Additional file 3: **Table S3.** Correlation indices between different samples. (XLSX 9 kb)

Additional file 4: **Figure S2.** Correlation indices between different samples (PDF 4 kb)

Additional file 5: **Table S4.** Summary of functional annotation of contigs from BLAST searches against public databases. (DOCX 12 kb)

Additional file 6: **Figure S3.** Homologous species of *Isodon amethystoides* transcriptomes. (TIF 110 kb)

Additional file 7: **Table S5.** Transcription factors identified in this study. (XLSX 116 kb)

Additional file 8: **Figure S4.** Distribution of transcription factor families. (TIF 1288 kb)

Additional file 9: **Figure S5.** Frequencies and mean expression levels of transcripts matching GO terms. The percentage of transcripts matching GO terms is shown for each category. (PNG 139 kb)

Additional file 10: **Table S6.** GO enrichment of unigenes. (XLS 8009 kb)

Additional file 11: **Table S7.** Unigenes for KEGG analysis. (XLSX 667 kb)

Additional file 12: **Table S8.** DEGs between root, stem and leaf. (XLSX 2009 kb)

Additional file 13: **Table S9.** KEGG enrichment analysis list. (XLS 73 kb)

Additional file 14: **Table S10.** Summary of transcripts in *Isodon amethystoides* encoding enzymes involved in the tetracyclic terpenoid biosynthesis. (DOCX 22 kb)

Additional file 15: **Table S1.** The primers used for qRT-PCR. (DOC 34 kb)

## Abbreviations

CPS: *ent-*copalyl diphosphate synthase
DEGs: Differentially expressed genes
DMAPP: Dimethylallyl diphosphate
DWT: Dry weight
DXR: 1-deoxy-d-xylulose-5-phosphate reductoisomerase
FPKM: Fragments per kilobase per million reads
GGPP: Geranyl geranyl-PP
IPP: Isopentenyl diphosphate
ISPD: 2-C-methyl-d-erythritol 4-phosphate cytidylyltransferase
ISPF: 2C-Methyl-D-erythritol 2,4-cyclodiphosphate synthase
ISPG: 4-hydroxy-3-methylbut-2-en-1-yl diphosphate synthase
ISPH: 1-hydroxy-2-methyl-2-(E)-butenyl 4-diphosphate reductase
KAO: *ent-*kaurenoic acid oxidase
KOS: *ent-*kaurene oxidase
KSL: *ent-*kaurene synthase-like
MEP: Methylerythritol phosphate
qRT-PCR: Quantitative reverse transcription PCR
