# Supplementary material for: Comparative transcriptome analysis of roots, stems and leaves of Isodon amethystoides reveals candidate genes involved in Wangzaozins biosynthesis
Source: BMC Plant Biol. 2018 Nov 8;18:272. doi: 10.1186/s12870-018-1505-0 (PMC6225716; doi:10.1186/s12870-018-1505-0)
Supplement: Supplementary file 1 — Table S2. Overview of transcriptome sequencing and de novo assembly. (DOC 18 kb) [file 12870_2018_1505_MOESM1_ESM.doc]

**Supplementary Table S2.** Overview of transcriptome sequencing and *de novo*
assembly results

|  | Root | Stem | Leaf |
| --- | --- | --- | --- |
| Clean data | 7142179300 | 7192855600 | 7425602000 |
| Q30 percentage | 89.68% | 90.63% | 90.56% |
| Clean reads | 71421793 | 71928556 | 74256019 |
|  |  |  |  |
| Total transcripts | 114488 | | |
| Mean length | 858 | | |
| N50 | 1241 | | |
| Percentage GC | 47.43% | | |
| Total bases | 279335919 | | |
