# Supplementary figures and images for: Comparative transcriptome analysis of roots, stems and leaves of Isodon amethystoides reveals candidate genes involved in Wangzaozins biosynthesis
Source: BMC Plant Biol. 2018 Nov 8;18:272. doi: 10.1186/s12870-018-1505-0 (PMC6225716; doi:10.1186/s12870-018-1505-0)

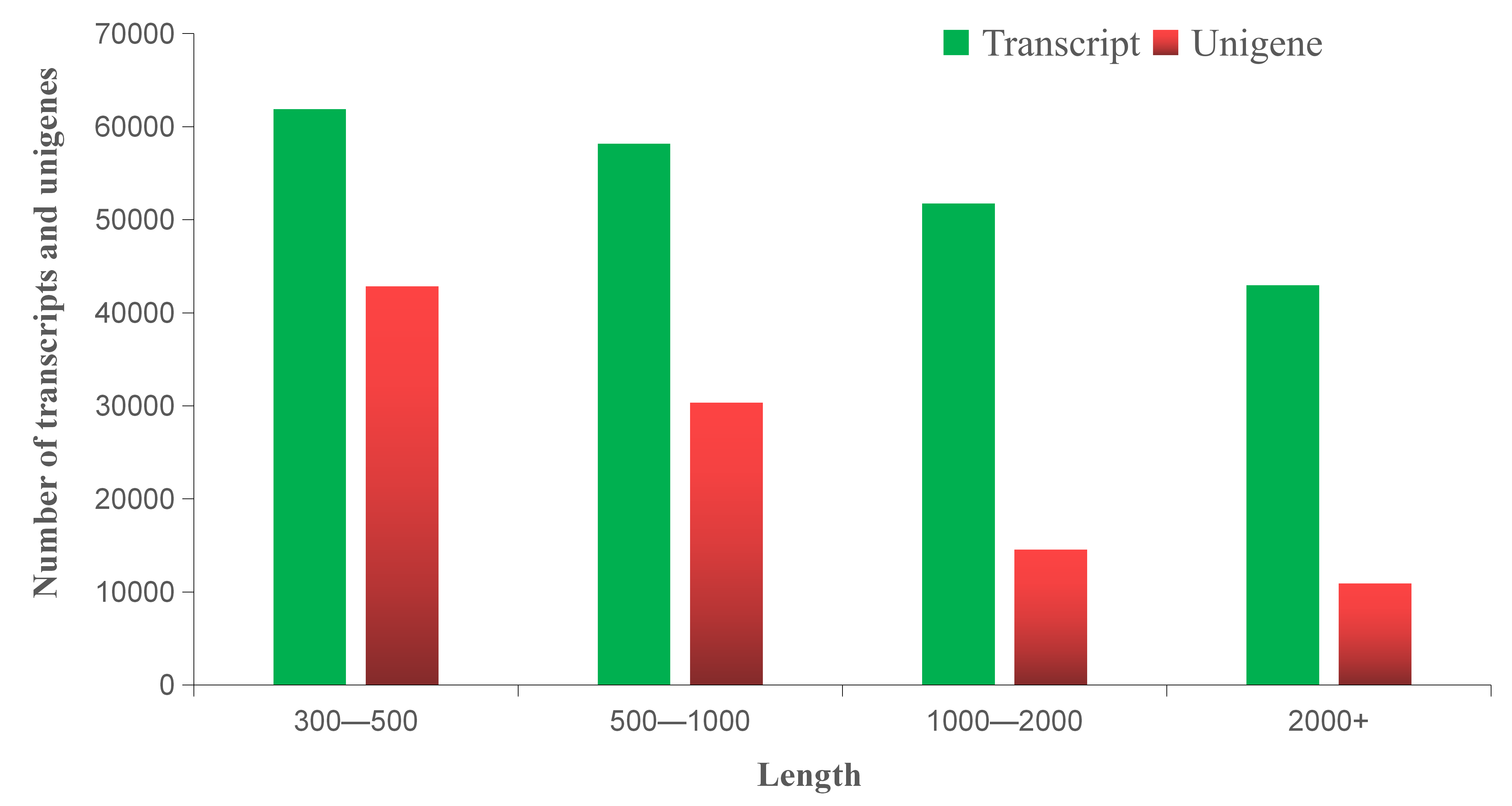

Supplement: Supplementary file 2 — Figure S1. Overview of transcriptome assembly showing size distribution. (TIF 714 kb) [file 12870_2018_1505_MOESM2_ESM.tif]

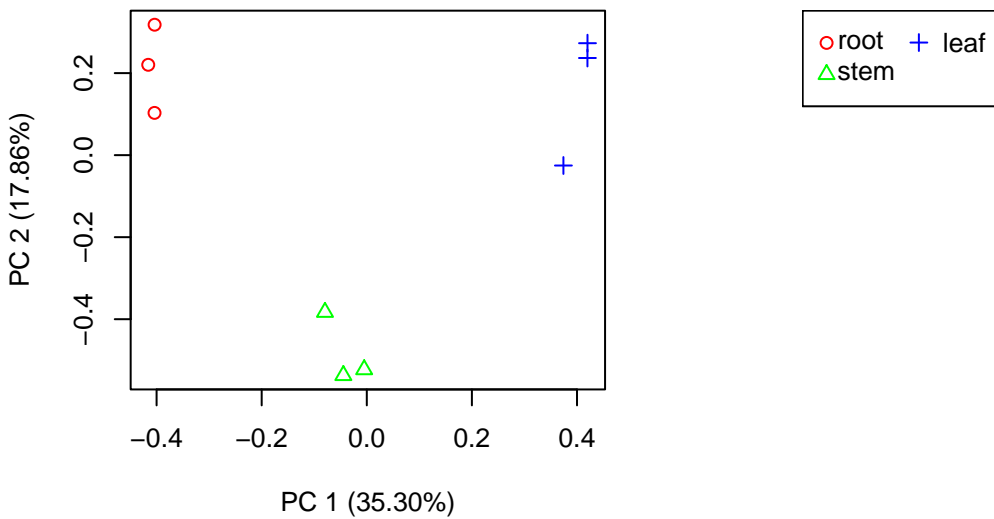

Supplement: Supplementary file 4 — Figure S2. Correlation indices between different samples (PDF 4 kb) [file 12870_2018_1505_MOESM4_ESM.pdf]

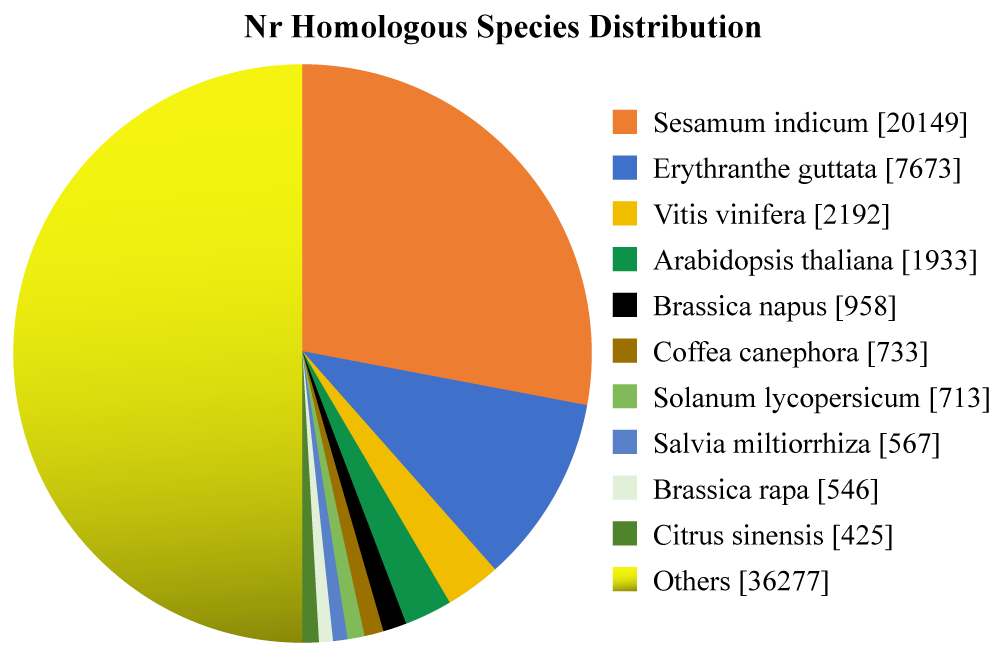

Supplement: Supplementary file 6 — Figure S3. Homologous species of Isodon amethystoides transcriptomes. (TIF 110 kb) [file 12870_2018_1505_MOESM6_ESM.tif]

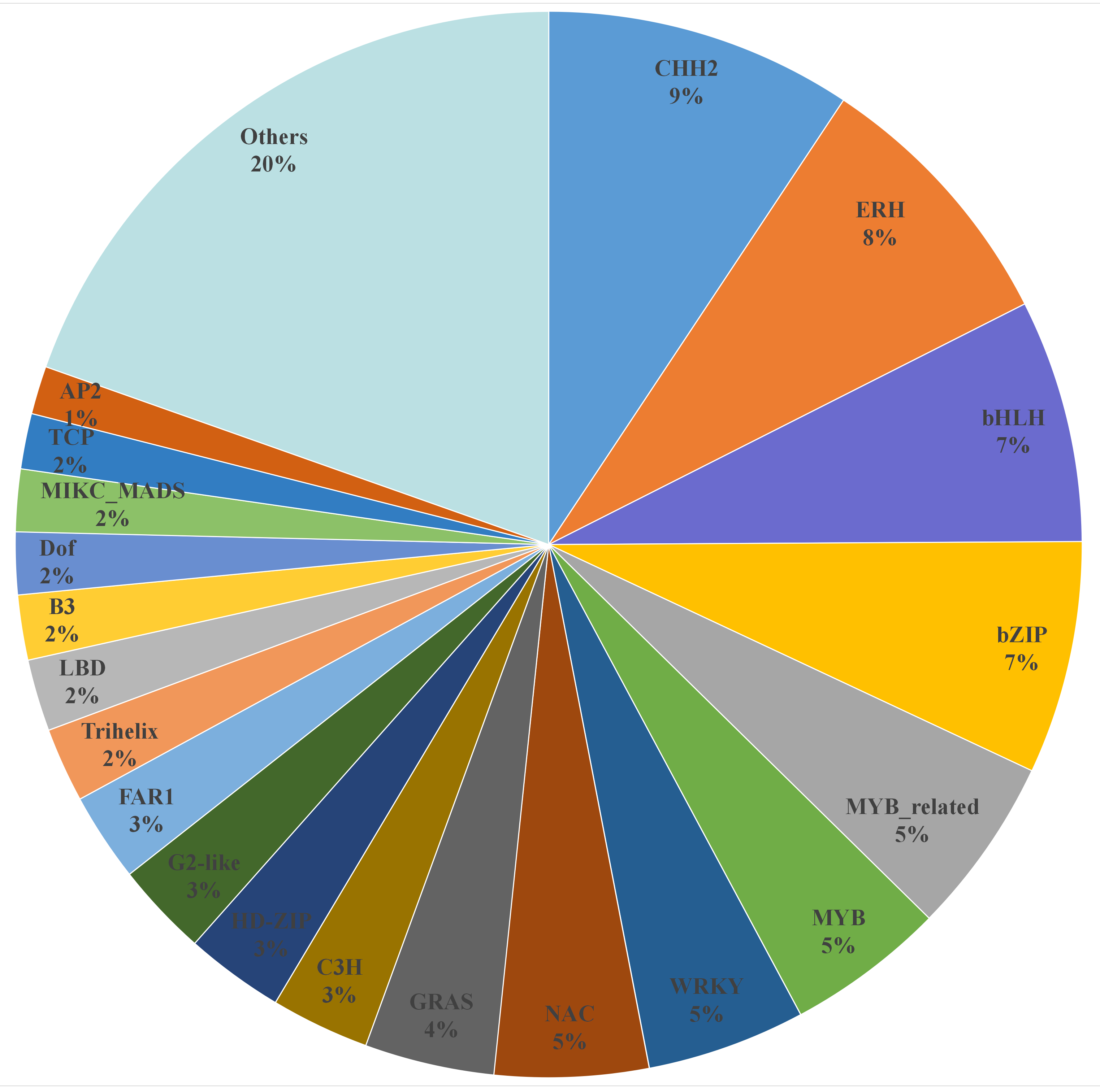

Supplement: Supplementary file 8 — Figure S4. Distribution of transcription factor families. (TIF 1288 kb) [file 12870_2018_1505_MOESM8_ESM.tif]

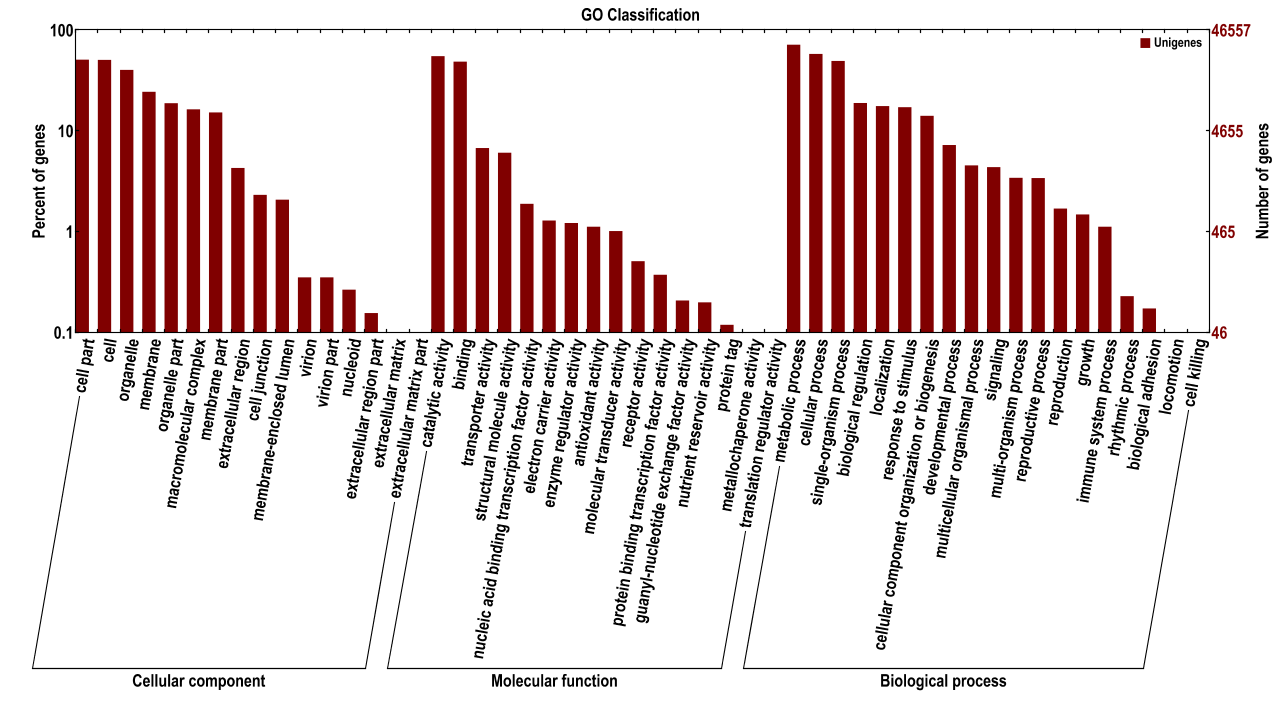

Supplement: Supplementary file 9 — Figure S5. Frequencies and mean expression levels of transcripts matching GO terms. The percentage of transcripts matching GO terms is shown for each category. (PNG 139 kb) [file 12870_2018_1505_MOESM9_ESM.png]
