# Supplementary material for: Comparative transcriptome analysis of roots, stems and leaves of Isodon amethystoides reveals candidate genes involved in Wangzaozins biosynthesis
Source: BMC Plant Biol. 2018 Nov 8;18:272. doi: 10.1186/s12870-018-1505-0 (PMC6225716; doi:10.1186/s12870-018-1505-0)
Supplement: Supplementary file 5 — Table S4. Summary of functional annotation of contigs from BLAST searches against public databases. (DOCX 12 kb) [file 12870_2018_1505_MOESM5_ESM.docx]

**Supplementary Table S4.** Summary of functional annotation of contigs from BLAST searches against public databases

| Annotated databases | Unigene | ≥300 bp | ≥1000 bp |
| --- | --- | --- | --- |
| COG | 25101 | 15504 | 9597 |
| GO | 46557 | 30722 | 15835 |
| KEGG | 17527 | 11918 | 5609 |
| KOG | 40517 | 26266 | 14251 |
| Pfam | 47026 | 28158 | 18868 |
| Swissprot | 47100 | 29060 | 18040 |
| NR | 72165 | 48789 | 23376 |
| All | 73693 | 50163 | 23530 |
