# Supplementary material for: Comparative transcriptome analysis of roots, stems and leaves of Isodon amethystoides reveals candidate genes involved in Wangzaozins biosynthesis
Source: BMC Plant Biol. 2018 Nov 8;18:272. doi: 10.1186/s12870-018-1505-0 (PMC6225716; doi:10.1186/s12870-018-1505-0)
Supplement: Supplementary file 14 — Table S10. Summary of transcripts in Isodon amethystoides encoding enzymes involved in the tetracyclic terpenoid biosynthesis. (DOCX 22 kb) [file 12870_2018_1505_MOESM14_ESM.docx]

**Supplementary Table S10.** Summary of transcripts in *Isodon amethystoides* encoding enzymes involved in the tetracyclic terpenoid biosynthesis

| **Pathway** | **Gene** | **E.C. number** | | **KO** | **ID number** | **Best match (NCBI GenBank)** | **AA identity** |
| --- | --- | --- | --- | --- | --- | --- | --- |
| MEP | IaDXS1 | 2.2.1.7 | K01662 | | DN57306_c0_g1 | DXS Isodon rubescens [AMM72794.1] | 99% |
|  | IaDXS2 | 2.2.1.7 | K01662 | | DN54153_c0_g1 | putative DXS, Salvia miltiorrhiza [AEZ55686.1] | 88% |
|  | IaDXS3 | 2.2.1.7 | K01662 | | DN60486_c0_g1 | Putative DXS  precursor, Salvia miltiorrhiza [AEZ55687.1] | 89% |
|  | IaDXS4 | 2.2.1.7 | K01662 | | DN64453_c1_g3 | probable DXS, chloroplastic, Sesamum indicum [XP_011090872] | 87% |
|  | IaDXR1 | 1.1.1.267 | K00099 | | DN59951_c0_g1 | DXR, Isodon rubescens[AMW77343.1] | 90% |
|  | IaDXR2 | 1.1.1.267 | K00099 | | DN59951_c0_g2 | DXR, Morus notabilis[EXB94935.1] | 72% |
|  | IaISPD | 2.7.7.60 | K00991 | | DN59073_c0_g1 | ispD, Salvia miltiorrhiza  [ AEZ55666.1] | 81% |
|  | IaISPE1 | 2.7.1.148 | K00919 | | DN63244_c2_g2 | ispE, Ginkgo biloba [AAZ80384.1] | 72% |
|  | IaISPE2 | 2.7.1.148 | K00919 | | DN63244_c2_g4 | ispE, Plectranthus barbatus  [[AHG98062.1](https://www.ncbi.nlm.nih.gov/protein/AHG98062.1?report=genbank&log$=protalign&blast_rank=1&RID=N3YGU1N5015)] | 94% |
|  | IaISPF | 4.6.1.12 | K01770 | | DN53905_c0_g3 | ispF, Isodon rubescens [AMW77341.1] | 94% |
|  | IaISPG1 | 1.17.7.1 | K03526 | | DN49525_c0_g2 | ispG, Brassica napus [[XP_022565354.1](https://www.ncbi.nlm.nih.gov/protein/XP_022565354.1?report=genbank&log$=protalign&blast_rank=1&RID=N3YZASJR015)] | 99% |
|  | IaISPG2 | 1.17.7.1 | K03526 | | DN49525_c0_g1 | Brassica oleracea var. oleracea [[XP_013608333.1](https://www.ncbi.nlm.nih.gov/protein/XP_013608333.1?report=genbank&log$=protalign&blast_rank=4&RID=N3Z90MG5014)] | 99% |
|  | IaISPH | 1.17.1.2 | K03527 | | DN83873_c0_g1 | IspH, Solanum lycopersicum [[XP_015056327.1](https://www.ncbi.nlm.nih.gov/protein/XP_015056327.1?report=genbank&log$=protalign&blast_rank=1&RID=N3ZGARMH014)] | 98% |
|  | IaIDI | 5.3.3.2 | K01823 | | DN85218_c0_g1 | IaIDI, Equus przewalskii | 94% |
| Tetracyclic diterpenoid | IaGPPS1 | 2.5.1.1 | K14066 | | DN54001_c0_g1 | GPPS small subunit, Salvia miltiorrhiza  [AEZ55680.1] | 91% |
|  | IaGPPS2 | 2.5.1.1 | K14066 | | DN56749_c0_g1 | GPPS large subunit， Mentha x piperita  [AAF08793.1] | 78% |
|  | IaGGPPS1 | 2.5.1.29 | K13789 | | DN49479_c0_g1 | GGPPS 3, Leucosceptrum canum  [ALT16903.1] | 79% |
|  | IaGGPPS2 | 2.5.1.29 | K13789 | | DN57201_c0_g1 | GGPPS 2, Salvia miltiorrhiza  [[AEZ55682.1](https://www.ncbi.nlm.nih.gov/protein/AEZ55682.1?report=genbank&log$=protalign&blast_rank=2&RID=N41PBZNK01R)] | 72% |
|  | IaGGPPS3 | 2.5.1.29 | K13789 | | DN42614_c0_g1 | GGPPS 7, chloroplastic-like ,Olea europaea var. sylvestris  [XP_022845144.1] | 83% |
|  | IaGGPPS4 | 2.5.1.29 | K13789 | | DN48026_c0_g1 | GGPPS small subunit Lavandula x intermedia]  [AGH33891.1] | 67% |
|  | IaCPS1 | 5.5.1.13 | K04120 | | DN64742_c2_g3 | ent-copalyl diphosphate synthase, Isodon rubescens  [ARO38143.1] | 97% |
|  | IaCPS2 | 5.5.1.13 | K04120 | | DN53207_c0_g1 | ent-copalyl diphosphate synthase, Isodon eriocalyx  [AEP03177.1] | 98% |
|  | IaCPS3 | 4.2.1.133 | K04120 | | DN34471_c0_g1 | copalyl diphosphate synthase 3,Isodon rubescens  [ APJ36373.1] | 98% |
|  | IaCPS4 | 4.2.1.133 | K04120 | | DN52118_c0_g1 | copalyl diphosphate synthase 1,Isodon rubescens  [APJ36371.1] | 90% |
|  | IaCPS5 | 4.2.1.133 | K04120 | | DN61890_c0_g1 | copalyl diphosphate synthase,Salvia rosmarinus  [ AHL67261.1] | 81% |
|  | IaKSL1 | 4.2.3.19 | K04121 | | DN62357_c0_g2 | kaurene synthase 4, Isodon rubescens [ASC55316.1] | 95% |
|  | IaKSL2 | 4.2.3.19 | K04121 | | DN63495_c2_g2 | kaurene synthase 5, Isodon rubescens [[ASC55317.1](https://www.ncbi.nlm.nih.gov/protein/ASC55317.1?report=genbank&log$=protalign&blast_rank=1&RID=N47PC1SW01R)] | 98% |
|  | IaKSL3 | 4.2.3.19 | K04121 | | DN62357_c0_g3 | kaurene kaurene synthase 4, Isodon rubescens [ASC55316.1] | 86% |
|  | IaKSL4 | 4.2.3.19 | K04121 | | DN63900_c1_g6 | kaurene synthase 4, Isodon rubescens [ASC55316.1] | 95% |
|  |  |  |  | |  |  |  |

DXS: 1-deoxy-d-xylulose-5-phosphate synthase; DXR: 1-deoxy-d-xylulose-5-phosphate reductoisomerase; ISPD: 2-C-methyl-d-erythritol 4-phosphate cytidylyltransferase; ISPE: 4-diphosphocytidyl-2-C-methyl-d-erythritol kinase; ISPF: 2-C-methyl-d-erythritol 2,4-cyclodiphosphate synthase; ISPG: 4-hydroxy-3-methylbut-2-en-1-yl diphosphate synthase; ISPH: 1-hydroxy-2-methyl-2-(E)-butenyl 4-diphosphate reductase; IDI: isopentenyl-diphosphate delta-isomerase; GPPS: geranyl diphosphate synthase; GGPS: geranyl geranyl diphosphate synthase; CPS: ent-copalyl diphosphate synthase/copal-8-ol diphosphate hydratase; KSL: ent-kaur-16-ene synthase-like.
