# Supplementary material for: Comparative transcriptome analysis of roots, stems and leaves of Isodon amethystoides reveals candidate genes involved in Wangzaozins biosynthesis
Source: BMC Plant Biol. 2018 Nov 8;18:272. doi: 10.1186/s12870-018-1505-0 (PMC6225716; doi:10.1186/s12870-018-1505-0)
Supplement: Supplementary file 15 — Table S1. The primers used for qRT-PCR. (DOC 34 kb) [file 12870_2018_1505_MOESM15_ESM.doc]

**Supplementary Table S1** Primers used for qRT-PCR

| Gene ID | Name | Amplicon |  | Sequences |
| --- | --- | --- | --- | --- |
| DN63900_c1_g6 | IaKSL4 | 102 bp | Forward | CAGCACTTAAGCGCACAATC |
|  |  |  | Reverse | CAGCAGATCCAACCAAATG |
|  |  |  |  |  |
| DN53905_c0_g3 | IaISPF | 114 bp | Forward | TCACTCCGATGGTGATGTATTG |
|  |  |  | Reverse | GCTCCTTTCCACTTAGGATCTG |
|  |  |  |  |  |
| DN57306_c0_g1 | IaDXS1 | 107 bp | Forward | GTAGATGGCCACAGTATGGAAG |
|  |  |  | Reverse | CCTTTGCCCTTCTCGGTTAT |
|  |  |  |  |  |
| DN59951_c0_g1 | IaDXR1 | 107 bp | Forward | GCTGCTAGTGCAACGATTCTA |
|  |  |  | Reverse | GGTCCGAAGCCTATTTCAGTTAT |
|  |  |  |  |  |
| DN59073_c0_g1 | IaISPD | 111 bp | Forward | CACCAGTAGTGGTTGAAGAGAA |
|  |  |  | Reverse | GTTGGCCAAGTAGTGGAAGATA |
|  |  |  |  |  |
| DN83873_c0_g1 | IaISPH | 115 bp | Forward | CATCCATATCCTCTAGCCTCTTATTC |
|  |  |  | Reverse | CGTGCAGTTCAGATTGCTTATG |
|  |  |  |  |  |
| DN57201_c0_g1 | IaGGPS2 | 148 bp | Forward | TTGGGAGAATATGGTCGGATTG |
|  |  |  | Reverse | CTTTCTTAGCCCGAGACTTGAG |
|  |  |  |  |  |
| DN64742_c2_g3 | IaCPS1 | 100 bp | Forward | GATGTGGTGTCAGAGAAGATGAG |
|  |  |  | Reverse | CAGTGTCGTACGGCGATATG |
|  |  |  |  |  |
| DN64788_c2_g1 | IaActin | 118 bp | Forward | CAACCGATCGTATGCGACAA |
|  |  |  | Reverse | CCATCACGCCTCGGTATTTG |
